# Change in Depressive Symptoms and Longitudinal Regional Amyloid Accumulation in Unimpaired Older Adults

**DOI:** 10.1001/jamanetworkopen.2024.27248

**Published:** 2024-08-29

**Authors:** Catherine E. Munro, Michelle Farrell, Bernard Hanseeuw, Dorene M. Rentz, Rachel Buckley, Michael Properzi, Ziwen Yuan, Patrizia Vannini, Rebecca E. Amariglio, Yakeel T. Quiroz, Deborah Blacker, Reisa A. Sperling, Keith A. Johnson, Gad A. Marshall, Jennifer R. Gatchel

**Affiliations:** 1 Brigham and Women’s Hospital, Harvard Medical School, Boston, Massachusetts; 2 Massachusetts General Hospital, Harvard Medical School, Boston; 3 Athinoula A. Martinos Center for Biomedical Imaging, Charlestown, Massachusetts; 4 Institute of Neuroscience, Université Catholique de Louvain/Cliniques Universitaires Saint-Luc, Brussels, Belgium; 5 Grupo de Neurociencias de Antioquia, Universidad de Antioquia, Medellin, Colombia; 6 Department of Epidemiology, Harvard T.H. Chan School of Public Health, Boston, Massachusetts; 7 McLean Hospital, Belmont, Massachusetts; 8 Baylor College of Medicine, Houston, Texas; 9 Michael E. Debakey Department of Veterans Affairs Medical Center, Houston, Texas

## Abstract

**Importance:**

Depressive symptoms in older adults may be a harbinger of Alzheimer disease (AD), even in preclinical stages. It is unclear whether worsening depressive symptoms are manifestations of regional distributions of core AD pathology (amyloid) and whether cognitive changes affect this relationship.

**Objective:**

To evaluate whether increasing depressive symptoms are associated with amyloid accumulation in brain regions important for emotional regulation and whether those associations vary by cognitive performance.

**Design, Setting, and Participants:**

Participants from the Harvard Aging Brain Study, a longitudinal cohort study, underwent annual assessments of depressive symptoms and cognition alongside cortical amyloid positron emission tomography (PET) imaging at baseline and every 2 to 3 years thereafter (mean [SD] follow-up, 8.6 [2.2] years). Data collection was conducted from September 2010 to October 2022 in a convenience sample of community-dwelling older adults who were cognitively unimpaired with, at most, mild baseline depression. Data were analyzed from October 2022 to December 2023.

**Main Outcomes and Measures:**

Depression (Geriatric Depression Scale [GDS]–30-item), cognition (Preclinical Alzheimer Cognitive Composite–5 [PACC]), and a continuous measure of cerebral amyloid (Pittsburgh compound B [PiB] PET) examined in a priori–defined regions (medial orbitofrontal cortex [mOFC], lateral orbitofrontal cortex, middle frontal cortex [MFC], superior frontal cortex, anterior cingulate cortex, isthmus cingulate cortex [IC], posterior cingulate cortex, and amygdala). Associations between longitudinal GDS scores, regional amyloid slopes, and PACC slopes were assessed using linear mixed-effects models.

**Results:**

In this sample of 154 individuals (94 [61%] female; mean [SD] age, 72.6 [6.4] years; mean (SD) education, 15.9 [3.1] years), increasing PiB slopes in the bilateral mOFC, IC, and MFC were associated with increasing GDS scores (mOFC: β = 11.07 [95% CI, 5.26-16.87]; *t* = 3.74 [SE, 2.96]; *P* = .004; IC: β = 12.83 [95% CI, 5.68-19.98]; *t* = 3.51 [SE, 3.65]; *P* = .004; MFC: β = 9.22 [95% CI, 2.25-16.20]; *t* = 2.59 [SE, 3.56]; *P* = .03). Even with PACC slope as an additional covariate, associations remained significant in these regions.

**Conclusions and Relevance:**

In this cohort study of cognitively unimpaired older adults with, at most, mild baseline depressive symptoms, greater depressive symptoms over time were associated with amyloid accumulation in regions associated with emotional control. Furthermore, these associations persisted in most regions independent of cognitive changes. These results shed light on the neurobiology of depressive symptoms in older individuals and underscore the importance of monitoring for elevated mood symptoms early in AD.

## Key Points

**Question:**

Are emerging depressive symptoms in cognitively unimpaired older adults associated with early regional amyloid accumulation?

**Findings:**

In this cohort study using longitudinal data from 154 older adults who were cognitively unimpaired and had mild or no depressive symptoms at baseline, increasing depressive symptoms were associated with early amyloid accumulation in brain regions involved in emotional control, independent of cognitive performance.

**Meaning:**

These findings suggest that increasing depressive symptoms in older adults may be associated with increasing regional Alzheimer disease pathology, which has implications for recognizing individuals in preclinical Alzheimer disease stages who may be candidates for disease-modifying treatments and for considering depression treatment to prevent dementia.

## Teaser

This cohort study examines whether depressive symptoms are associated with amyloid accumulation in the brains of community-dwelling older adults with no cognitive impairment at baseline.

## Introduction

Neuropsychiatric symptoms (NPS), particularly depression, are common in Alzheimer disease (AD).^1,2^ Recent research has linked elevated depressive symptomatology in part to AD pathophysiology, even in preclinical stages of AD (ie, before cognitive impairment), including greater core AD pathology burden, changes in functional connectivity, and cerebral metabolic dysfunction.^3,4,5,6,7,8,9,10,11^

While growing evidence supports associations between emerging depressive symptoms and preclinical AD, most work has focused on cross-sectional data with large-scale measures of brain function or pathophysiology (ie, large cortical aggregates of amyloid burden or large-scale functional networks). However, studies examining clinical AD stages have found associations between depression and regional distributions of AD-related biomarkers. For example, a review of mechanisms underlying NPS in AD using lesion patterns^12^ found that AD-related depression was consistently associated with the frontal-limbic circuit, encompassing areas necessary for emotional control. Similarly, a recent study^13^ found that more severe NPS, including depression, in preclinical individuals was not only associated with shorter time-to–dementia-onset but also greater cerebral metabolic decline in mood-related regions, including the posterior cingulate and prefrontal cortices. Furthermore, not all studies have consistently defined the nature of depressive symptoms, particularly whether they stem from a recurrent depressive illness, are a psychological reaction to insight about cognitive decline, or emerge in conjunction with cognitive changes in early stages of neurodegenerative diseases.

Depressive symptoms have also been linked to cognitive, functional, and pathophysiological (ie, amyloid, tau burden) changes in cognitively unimpaired individuals. Prior work from our group^14^ found that baseline cortical amyloid levels moderated the association between increasing depressive symptoms and worsening cognition in cognitively unimpaired older adults. Another group^15^ showed that cognitively unimpaired participants with greater NPS (most commonly, depression) and abnormalities in cerebral spinal fluid markers of amyloid and tau demonstrated more rapid progression of functional impairment than those without NPS. However, more work is needed to better understand trajectories of depressive symptoms in cognitively unimpaired older adults and early amyloid accumulation in the context of cognitive decline. To fill this gap in the literature, the current study seeks to examine longitudinal depressive symptoms and early regional amyloid accumulation in the context of change in objective cognitive performance. We aimed to do this in a unique cohort of older adults who, at study entry (1) were cognitively unimpaired; (2) had, at most, mild depressive symptoms; and (3) had initially low global cortical amyloid burden. We did this to capture not only overall deposition but also early regional accumulation over time, well before the stage of cortical amyloid positivity that is present at stages of impairment (ie, mild cognitive impairment [MCI] and dementia).

Utilizing a sample of participants with low cortical amyloid at baseline has several advantages for understanding these associations. First, this sample allows us to learn whether early changes in depressive symptomatology accompany the earliest accumulation of amyloid in the brain, similar to what has been previously found regarding cognitive decline and subthreshold amyloid accumulation.^16,17^ This could provide crucial information about the possible temporal concurrence of initial changes in pathology and mood. Additionally, from a methodological perspective, evidence suggests accumulation of amyloid in the brain may be nonlinear over time, with accumulation plateauing after several decades.^18^ This can affect our ability to model amyloid accumulation in individuals with elevated amyloid levels at study baseline. Studying a population with low cortical amyloid at baseline over 8 years of mean follow-up minimizes the potential effect of plateauing, making the amyloid slope a more meaningful factor in the analysis.

Furthermore, to better understand the neurobiology of AD-related depressive symptoms, we sought to examine longitudinal associations among depression, cognition, and amyloid in specific regions of interest (ROIs) linked to affective regulation that are also commonly affected by AD pathology. Key ROIs in the frontal and cingulate cortices were identified a priori, including the medial and lateral orbitofrontal cortices, the middle frontal and superior frontal gyri (representing regions of the dorsal-lateral prefrontal cortex), and the anterior cingulate cortex, as these regions are thought to be important for emotional salience and have been linked to the pathophysiology of major depressive disorder.^19,20,21^ Additional ROIs included the isthmus and posterior cingulate cortices, which have been linked to depressive mood states and somatic depressive symptoms.^22^ The final ROI was the amygdala, a limbic system component important for fear and stress, as changes in amygdala volume have been linked to depressive disorders.^19,23^ Although the amygdala often has low amyloid accumulation in preclinical stages, it was included based on its importance to mood. All ROIs have also been linked to AD pathology at different stages; limbic system regions can be affected early in the AD continuum, but pathology tends to spread to prefrontal and cingulate regions over time.^24^

The main objective of this study was to determine whether longitudinal depressive symptoms were associated with longitudinal regional amyloid accumulation in key ROIs related to emotional control (ie, medial orbitofrontal cortex [mOFC], lateral orbitofrontal cortex [lOFC], superior front cortex [SFC], middle frontal cortex [MFC], anterior cingulate cortex [ACC], posterior cingulate cortex [PCC], isthmus cingulate cortex [IC], and the amygdala) in cognitively unimpaired older adults with low amyloid and, at most, mild depressive symptoms at baseline. We hypothesized that increasing depressive symptoms over time would be associated with increasing amyloid levels in these ROIs.

Secondarily, we wanted to examine whether regional amyloid slopes remained associated with longitudinal depressive symptoms when adjusting for cognitive performance. While prior work from our group demonstrated associations between baseline global amyloid burden, longitudinal cognitive performance, and depressive symptoms,^14^ it is unclear whether these associations persist using longitudinal measurements of regional amyloid deposition and whether cognitive changes affect the association between longitudinal depressive symptoms and amyloid accumulation. We hypothesized that regional amyloid accumulation in ROIs related to emotional control would remain associated with increasing depressive symptoms, even after adjusting for cognitive changes.

Together, these objectives aim to help us better understand the earliest changes in depressive symptomatology alongside the earliest levels of amyloid accumulation, which could shed light on the neurobiology of depressive symptoms in preclinical AD. This may, in turn, assist with early clinical diagnosis and treatment for individuals eligible for anti-amyloid antibody therapy (eg, lecanemab, donanemab) to slow AD progression.

## Methods

### Study Sample

All participants were in the Harvard Aging Brain Study (HABS), a convenience cohort of cognitively unimpaired individuals aged 60 years and older at baseline.^25^ All participants had, at most, mild depressive symptoms at study entry (ie, 30-item Geriatric Depression Scale [GDS] scores <11).^26^ No cutoff criteria were set for follow-up GDS scores. Exclusion criteria included history of head trauma, unstable medical illness at recruitment, severe mental illness (eg, schizoaffective, bipolar disorders), or recent substance or alcohol abuse. Participants were permitted to have mild depression or anxiety at baseline or historically (per self-report), treated with a stable dose of serotonin, serotonin-norepinephrine reuptake inhibitors, bupropion, and/or low-dose nortriptyline.

All participants undergo extensive cognitive testing annually and multimodal neuroimaging, including amyloid (Pittsburgh compound B [PiB]–positron emission tomography [PET]) imaging, every 2 to 3 years. Consistent with other observational studies of cognitively unimpaired individuals, biomarker status is not disclosed to participants. Participants selected for these analyses (n = 154) were all below the cortical amyloid threshold of PiB-PET positivity at baseline. The present study followed the Strengthening the Reporting of Observational Studies in Epidemiology (STROBE) reporting guidelines for cohort studies (eMethods in Supplement 1). The study was approved by the institutional review board of Mass General Brigham. Written informed consent was obtained from all participants prior to initiation of any study procedures in accordance with IRB guidelines.

### Questionnaires and Neuropsychological Assessment

Depressive symptoms were assessed annually using the GDS long-form.^26^ This includes 30 yes-or-no questions designed to measure depressive symptomatology in older adults; scores of 0 to 10 represent no to mild depressive symptomatology; 11-19, mild to moderate; and 20-30, moderate to severe.

Objective cognitive performance was assessed annually using the Preclinical Alzheimer Cognitive Composite–5 (PACC), a composite of tests assessing episodic memory, executive functioning, and global cognition that is sensitive to early amyloid accumulation in preclinical AD.^27^ Ordinary least squares (OLS) regression slopes were calculated for longitudinal PACC data.

### Neuroimaging

Detailed magnetic resonance imaging (MRI)/PIB-PET acquisition parameters for HABS have been published previously^28,29,30,31,32,33,34,35,36,37^ (eMethods in Supplement 1). Distribution volume ratio (DVR) values were calculated via Logan plotting to estimate both amyloid burden within a standard large neocortical aggregate^25,28,38,39,40,41,42,43^ and amyloid burden in an a priori set of bilateral ROIs important for emotional control (ie, mOFC, lOFC, SFC, MFC, ACC, PCC, IC, and amygdala), defined from the Desikan-Killiany^44^ atlas via FreeSurfer version 6.0. OLS regression slopes were calculated separately for each ROI DVR. All participants fell below the PiB-PET detection threshold for global neocortical amyloid positivity at baseline (0.86 DVR) based on a common gaussian mixture modeling approach.^17,28^ Exploratory vertex-wise analyses were also conducted where similar mixed-effects models were run at each vertex using PiB slopes derived from OLS regression and effect size surfaces created for all fixed effects (eMethods in Supplement 1).

### Statistical Analysis

All analyses were conducted in R version 4.0.3 (R Project for Statistical Computing). The threshold for statistical significance was *P* < .05. For primary analyses, linear mixed-effects models were used to assess whether PiB slope × time was associated with longitudinal GDS score for each PiB ROI (ie, mOFC, lOFC, SFC, MFC, ACC, PCC, IC, and amygdala), adjusting for age, sex, education, and random intercept/slope (mathematical notation included in eMethods in Supplement 1). All *P* values were adjusted for multiple comparisons using an false-discovery rate correction. Models were repeated for each ROI including PACC slope × time as an additional covariate to adjust for cognitive change.

Sensitivity analyses were carried out to assess for potential confounds, including baseline amyloid levels, apolipoprotein E4 status, cognitive items on the GDS, self-reported history of depression and antidepressant use, and area deprivation index scores, a measure of multidimensional socioeconomic conditions.^45^ We also ran exploratory analyses examining 3-way interactions between regional PiB slopes, PACC slope, and time with longitudinal GDS scores for each PiB ROI to determine whether cognitive change moderated these associations (eMethods in Supplement 1).

## Results

A total of 154 participants, with a mean (SD) follow-up of 8.6 [2.2] years and 17 participants lost to follow up, included 94 (61%) female participants and 50 (39%) male participants with a mean baseline GDS score of 3.3 (2.9) (Table 1; eFigure 1 and eTable 1 in Supplement 1). The mean (SD) baseline PiB DVR of the sample using a large cortical aggregate was 0.8 (0.03) (using a longitudinal processing pipeline).

**Table 1.** Baseline Demographic and Clinical Characteristics of the 154 Participants in the Study Sample Included in Primary Analyses^a^

Demographic variable	Mean (SD) [range]
Age, y	72.6 (6.4) [61.0 to 89.3]
Sex, No. (%)	
Female	94 (61.0)
Male	60 (39.0)
Race, No. (%)	
American Indian or Alaska Native	1 (0.7)
Asian	1 (0.7)
Black	22 (14.3)
White	130 (84.4)
Ethnicity, No. (%)	
Hispanic	2 (1.3)
Non-Hispanic	152 (98.7)
Education, y	15.9 (3.1) [6.0 to 20.0]
ApoE ε4, No. (%)	42 (27.4)
History of depression, No. (%)	
Any	34 (22.4)
Within 10 y	27 (17.9)
Remote history	7 (4.4)
History of SSRI use, No. (%)	19 (12.6)
History of SNRI use, No. (%)	5 (3.0)
Baseline GDS score	3.3 (2.9) [0.0 to 12.0]
PiB DVR (frontolateral retrosplenial aggregate)	
Baseline	0.8 (0.03) [0.7 to 0.9]
Final	0.8 (0.04) [0.7 to 1.0]
PiB mOFC DVR	
Baseline	0.8 (0.04) [0.7 to 1.0]
Final	0.8 (0.07) [0.6 to 1.1]
PiB lOFC DVR	
Baseline	0.8 (0.06) [0.7 to 1.0]
Final	0.8 (0.06) [0.6 to 1.2]
PiB MFC DVR	
Baseline	0.8 (0.05) [0.06 to 0.9]
Final	0.8 (0.07) [0.7 to 1.0]
PiB SFC DVR	
Baseline	0.8 (0.04) [0.7 to 0.9]
Final	0.8 (0.06) [0.7 to 1.0]
PiB ACC DVR	
Baseline	0.9 (0.05) [0.8 to 1.0]
Final	0.9 (0.08) [0.7 to 1.2]
PiB IC DVR	
Baseline	0.9 (0.04) [0.8 to 1.0]
Final	0.9 (0.06) [0.8 to 1.1]
PiB PCC DVR	
Baseline	0.9 (0.04) [0.8 to 1.0]
Final	0.9 (0.06) [0.7 to 1.2]
PiB amygdala DVR	
Baseline	0.9 (0.04) [0.8 to 1.0]
Final	0.9 (0.05) [0.7 to 1.0]
PACC-5 *z* score	
Baseline	0.1 (0.7) [−1.8 to 2.1]
Final	0.1 (0.8) [−2.2 to 2.4]
Follow-up, y	8.6 (2.2) [2.8 to 11.1]

Abbreviations: ACC, anterior cingulate cortex; ApoE ε4, apolipoprotein E ε4 allele; DVR, distribution volume ratio; GDS, Geriatric Depression Scale; IC, isthmus cingulate cortex; lOFC, lateral orbitofrontal cortex; MFC, middle frontal cortex; mOFC, medial orbitofrontal cortex; PACC-5, Preclinical Alzheimer Cognitive Composite–5; PCC, posterior cingulate cortex; PiB, Pittsburgh Compound B; SFC, superior frontal cortex; SNRI, serotonin norepinephrine reuptake inhibitor; SSRI, selective serotonin reuptake inhibitor.

^a^ DVR values presented may appear slightly lower than DVR values calculated for traditional cross-sectional amyloid studies; the longitudinal processing pipeline utilizes an alternative composite reference region that is more stable over time compared with cross-sectional pipelines but tends to produce lower cortical and regional DVR values. The eMethods in Supplement 1 provides additional detail.

### Longitudinal Depressive Symptoms and Regional Amyloid Levels

**Figure 1.**
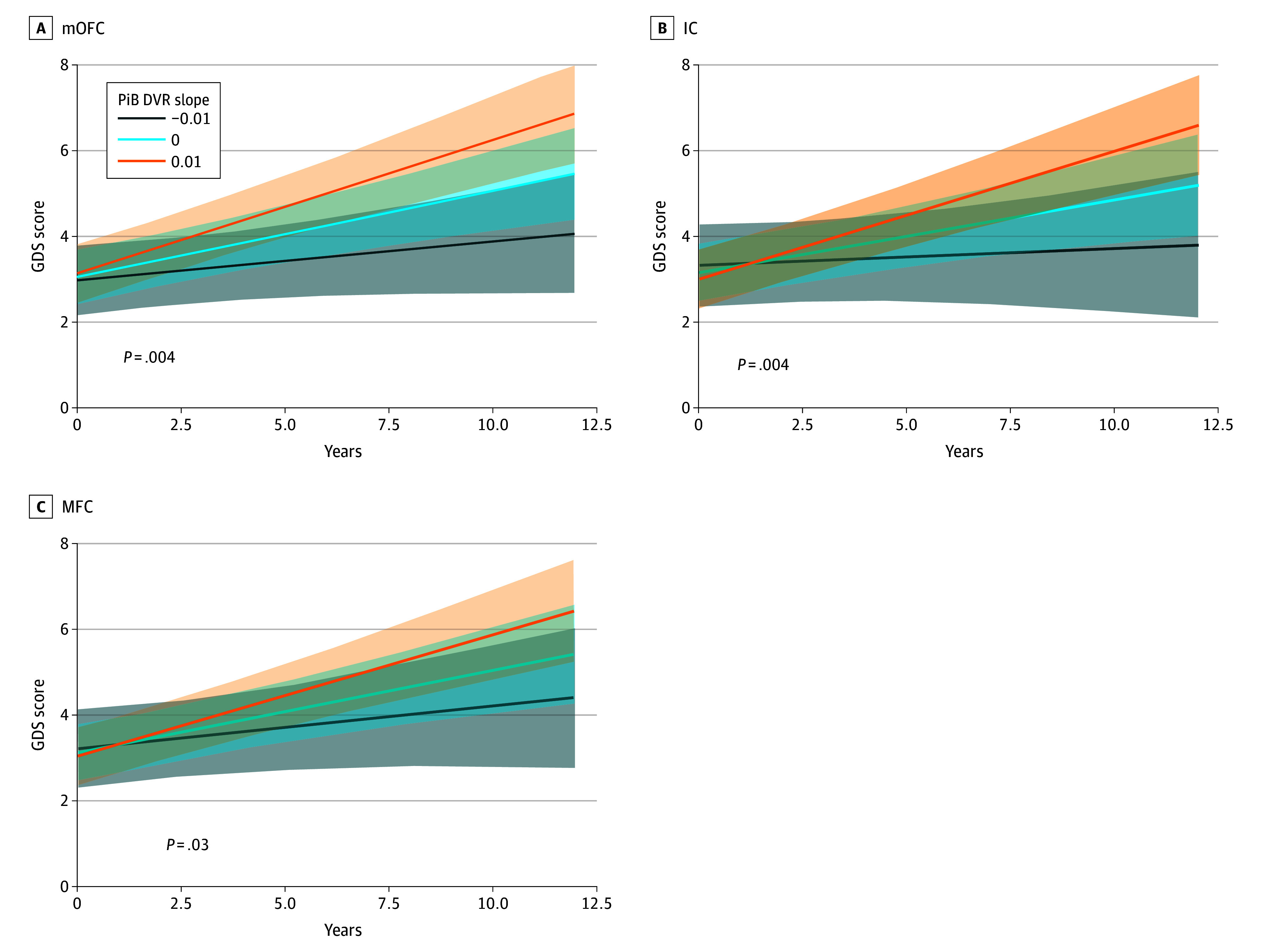
Geriatric Depression Scale (GDS) Score and Amyloid Accumulation in the Medial Orbitofrontal Cortex (mOFC), Isthmus Cingulate Cortex (IC), and Middle Frontal Cortex (MFC) Models for 154 patients showing association between GDS scores over time and amyloid slope (Pittsburgh Compound B distribution volume ratio [PiB DVR]) tertiles. The association in each region was statistically significant at *P* < .05, using *P* values adjusted with a false-discovery rate correction for multiple comparisons. Shaded areas indicate 95% CIs.

Increasing PiB (amyloid) slope over time was significantly associated with increasing GDS (depression score) over time in the mOFC (β = 11.07 [95% CI, 5.26-16.87]; *t* = 3.74 [SE, 2.96]; *P* = .004), IC (β = 12.83 [95% CI, 5.68-19.98]; *t* = 3.51 [SE, 3.65]; *P* = .004), and MFC (β = 9.22 [95% CI, 2.25-16.20]; *t* = 2.59 [SE = 3.56]; *P* = .03) (Table 2 and Figure 1). As visualized in Figure 1, a PiB DVR slope of 0.01 in the mOFC would be associated with an increase of approximately 1 point on the GDS per year. Amygdala, lOFC, SFC, ACC, and PCC PiB slopes were not associated with GDS score. Sex was not associated with GDS score in any models.

**Table 2.** Separate Linear Mixed-Effects Models for Longitudinal Geriatric Depression Scale Score and Amyloid Slope, Adjusting for Age, Sex, and Education

Region × time	β (95% CI)	*t* (SE)	Adjusted *P* value^a^
mOFC	11.07 (5.26 to 16.87)	3.74 (2.96)	.004
lOFC	3.01 (−2.17 to 8.19)	1.14 (2.64)	.35
MFC	9.22 (2.25 to 16.20)	2.59 (3.56)	.03
SFC	2.36 (−0.37 to −0.04)	0.69 (3.43)	.56
ACC	5.28 (0.21 to 10.36)	2.04 (2.59)	.08
IC	12.83 (5.68 to 19.98)	3.51 (3.65)	.004
PCC	4.06 (−1.85 to 9.98)	1.87 (3.01)	.29
Amygdala	−1.23 (−8.01 to 5.55)	−1.44 (3.46)	.72

Abbreviations: ACC, anterior cingulate cortex; IC, isthmus cingulate cortex; lOFC, lateral orbitofrontal cortex; MFC, middle frontal cortex; mOFC, medial orbitofrontal cortex; PCC, posterior cingulate cortex; SFC, superior frontal cortex.

^a^ *P* values following a false-discovery rate correction are reported.

### Longitudinal Depressive Symptoms, Regional Amyloid Levels, and Objective Cognitive Performance

Analyses including regional PiB slopes and PACC slope as independent variables showed that higher PiB slope in 3 regions was associated with increasing depression scores, even when adjusting for PACC slope: mOFC (β = 10.84 [95% CI, 5.22-16.46]; *t* = 3.78 [SE, 2.87]; *P* = .008), IC (β = 11.86 [95% CI, 4.88-18.85]; *t* = 3.33 [SE, 3.56]; *P* = .008), and MFC (β = 7.81 [95% CI, 0.95-14.66]; *t* = 2.23 [SE, 3.50]; *P* = .04) (Table 3). Lateral OFC, SFC, ACC, PCC, and amygdala PiB slopes were not significantly associated with depressive scores when adjusting for PACC slope. Sex was not significantly associated with depressive scores in any models.

**Table 3.** Separate Linear Mixed-Effects Models for Longitudinal Geriatric Depression Scale Score and Amyloid and PACC Slopes, Adjusting for Age, Sex, and Education

Independent variable (interaction with time) by region	β (95%CI)	*t* (SE)	Adjusted *P* value^a^
mOFC			
PiB slope	10.84 (5.22 to 16.46)	3.78 (2.87)	.008
PACC slope	−1.59 (−2.62 to −0.57)	−3.06 (0.52)	.008
lOFC			
PiB slope	2.38 (−2.66 to 7.42)	0.93 (2.57)	.41
PACC slope	−1.56 (−2.62 to −0.51)	−2.90 (0.54)	.008
MFC			
PiB slope	7.81 (0.95 to 14.66)	2.23 (3.50)	.04
PACC slope	−1.44 (−2.50 to −0.39)	−2.68 (0.54)	.01
SFC			
PiB slope	1.86 (−4.65 to 8.38)	0.56 (3.32)	.62
PACC slope	−1.59 (−2.65 to −0.54)	−2.96 (0.54)	.008
ACC			
PiB slope	4.67 (−0.27 to 9.62)	1.85 (2.52)	.09
PACC slope	−1.53 (−2.58 to −0.48)	−2.86 (0.53)	.008
IC			
PiB slope	11.86 (4.88 to 18.85)	3.33 (3.56)	.008
PACC slope	−1.47 (−2.51 to −0.44)	−2.79 (0.53)	.009
PCC			
PiB slope	3.05 (−2.71 to 8.81)	1.03 (2.94)	.36
PACC slope	−1.54 (−2.60 to −0.048)	−2.85 (0.54)	.008
Amygdala			
PiB slope	−0.34 (−6.94 to 6.26)	−0.10 (3.37)	.92
PACC slope	−1.60 (−6.94 to 6.26)	−2.96 (0.54)	.008

Abbreviations: ACC, anterior cingulate cortex; IC, isthmus cingulate cortex; lOFC, lateral orbitofrontal cortex; MFC, middle frontal cortex; mOFC, medial orbitofrontal cortex; PACC, Preclinical Alzheimer Cognitive Composite–5 Item; PCC, posterior cingulate cortex; PiB, Pittsburgh Compound B; SFC, superior frontal cortex.

^a^ *P* values following a false-discovery rate correction are reported.

### Vertex-Wise Maps

**Figure 2.**
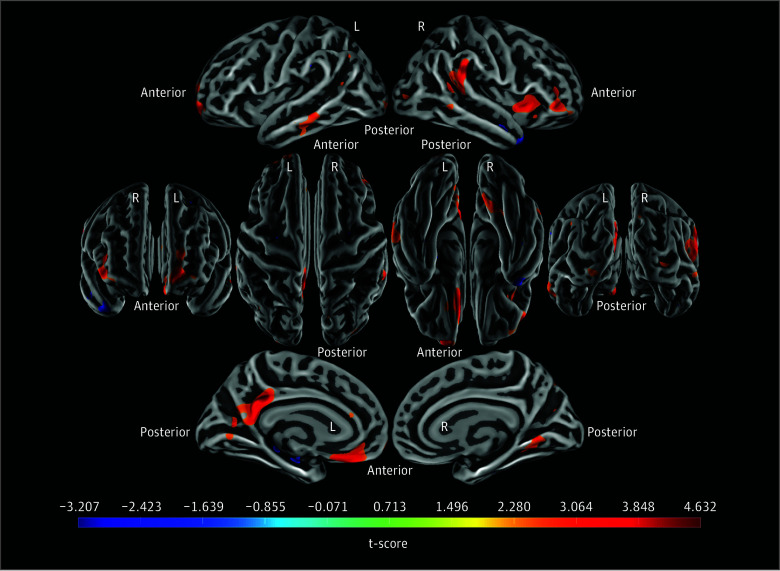
Vertex-Wise Map of Pittsburgh Compound B Distribution Volume Ratios (PiB DVR) PiB DVR images were projected onto fsaverage surfaces from FreeSurfer and surface smoothed, with PiB (amyloid) slope maps created using ordinary least squares regression (time as only factor) and mixed-effects models for 154 participants run at each vertex (*P* < .01; model: longitudinal Geriatric Depression Scale score estimated by amyloid slope × time + [sex + education + age] × time). This map visualizes the results of the linear mixed-effects models (*t* score of the main variable, amyloid slope × time) such that regions with a greater *t* score (red) represent areas in which greater depressive symptoms are associated with greater amyloid accumulation (amyloid slope). Anatomical landmarks are provided: L refers to the left hemisphere, R refers to the right hemisphere, and additional labels indicate the anterior and posterior ends of the brain.

The first vertex-wise map shows only PiB (amyloid) slope × time (time as only factor) vs longitudinal GDS scores (*P* < .01) (Figure 2). Regions with a greater *t* score (red) represent areas in which greater depressive symptoms are associated with greater amyloid levels. The second map additionally adjusts for PACC slope × time as a covariate (threshold *P* < .01) with similar results, featuring greater amyloid levels in frontal-cingulate areas associated with greater depressive symptoms but fewer highlighted lateral-frontal and parietal-occipital vertices compared with maps without PACC slope (eFigure 2 in Supplement 1).

### Sensitivity Analyses

Sensitivity analyses showed similar results to primary models, although depression history and antidepressant history did suppress the association seen between PACC slopes and GDS scores (eResults, eTables 3-7 in Supplement 1). Exploratory models were run using a 3-way interaction between PiB slopes, PACC slope, and time as the main factor. This 3-way interaction was significant when including PiB slopes in the MFC (β = −118.68 [95% CI, −190.82 to −46.54]; *t* = −3.23 [SE, 36.78]; *P* value = .008) and SFC (β = −118.68 [95% CI, −199.34 to −45.31]; *t* = −3.12 [SE, 39.27]; *P* = .008), indicating that the associations between longitudinal depressive symptoms and amyloid slope in these regions was moderated by cognitive change (eTable 2 in Supplement 1). This interaction of PACC slope × PiB slope × time was not significant in models using mOFC, lOFC, IC, PCC, ACC, and amygdala PiB slopes, although the covariate PiB slope × time in the mOFC and IC was significantly associated with GDS.

## Discussion

This study sought to examine longitudinal depressive symptomatology in relation to the earliest signs of regional accumulation of amyloid pathology in the brain over time in a cohort of cognitively unimpaired older adults who had, at most, mild depression at study baseline and were followed up for, on average, just under a decade. Results indicate that increasing depressive symptoms over time were associated with both increasing cortical amyloid levels in key brain regions related to mood and emotional control (ie, mOFC, MFC, and IC) and decreasing performance on objective cognitive measures over time. Additionally, associations between early amyloid accumulation in these regions (mOFC, MFC, and IC) and depressive symptoms over time remained significant, even after adjusting for longitudinal cognitive changes. Furthermore, cognitive change only modified the associations between regional amyloid accumulation and greater depressive symptoms in the MFC and SFC, while no 3-way interaction was seen in the mOFC and IC (only PiB slope × time remained a significant factor). While the effect sizes of the associations observed were small, they were within the magnitude of effect sizes we have observed in similar investigations in the HABS cohort and have important theoretical significance. This highlights that emerging depressive symptoms in older adults may not solely represent a psychological reaction stemming from insight into cognitive change but also might have a neurological basis localized to amyloid accumulation in frontal and cingulate cortices.

These results align with prior work linking new-onset symptoms of depression later in life with incident diagnosis of AD as well as AD-related cerebral pathophysiology (eg, amyloid).^46,47,48,49^ Adding to this literature, our findings, to our knowledge, are the first to examine in vivo, regional accumulation of one of the core components of AD pathology in older adults in preclinical disease stages using PET imaging. Results from another longitudinal cohort^49^ found that increasing GDS score was associated with an increased risk of incident dementia after 4.5 years, although the mechanisms underlying this increased risk have remained unclear. Additionally, a systematic review^48^ concluded depressive symptoms and dementia were related in a significant portion of patients studied, possibly due to common underlying risk factors and/or depression presenting as a prodromal dementia symptom (though some overlap between these factors is likely). Our findings provide additional support for depressive symptoms as an early feature of preclinical AD, while also supporting the potential role of other factors (eg, psychological) associated with at least some aspects of emerging depressive symptomatology. A review on depression as a target for preventing AD dementia^46^ details shared underlying biological mechanisms between depression and AD pathology, including changes in brain structure and function, inflammatory responses, and neurotransmitter imbalance. Our results support the idea that underlying AD pathology (eg, structural, inflammation, or excitotoxic damage from amyloid accumulation) could contribute to emerging depressive symptoms as a feature of early AD progression, in the preclinical disease stage, before objective cognitive impairment (ie, MCI, dementia).

### Limitations

This study has limitations. Our sample was a subset of the larger HABS cohort (those who had low amyloid at baseline) and is relatively small in relation to population-based or cohort studies and primarily composed of highly educated, White, non-Hispanic individuals. Thus, it will be critical in future work to examine associations in larger and more diverse samples, particularly as racially and ethnically minoritized individuals are at greater risk of developing dementia.^50^ Additionally, our sample was selected to have low overall burden of cerebrovascular disease at entry; further work should assess the potential impact of vascular disease on these associations and how depressive symptoms relate to risk for vascular dementia. It will also be important to examine associations relative to other biopsychosocial risk factors of depression and AD, including physical health, history of smoking or substance use, social relationships, and early-life trauma. So, too, will be examining other measures of affective symptoms; while the GDS was created to be sensitive to depression in older adults, prior work has shown that some items may better reflect other psychiatric symptomatology (eg, apathy, anxiety)^51^ or may reflect sequelae of aging (eg, sleep disruption, age-related cognitive decline). Prior work in both psychiatric and AD literature^14^ has shown sex differences in vulnerability for psychiatric illness and in associations between cognitive decline and AD pathology. While sex was not significant in our models, future, larger-scale analyses are needed to further probe associations among sex, gender, psychiatric symptomatology, and AD risk. Coupling this with analyses of circuit-level function (eg, regional structural MRI, white matter integrity, and brain networks using functional connectivity MRI) will be critical next steps in elucidating neurobiological mechanisms underlying mood symptoms in AD.

## Conclusions

In a cohort of cognitively unimpaired older adults with low cortical amyloid and, at most, mild depressive symptoms at baseline, increasing depressive symptoms over time were significantly associated with early amyloid accumulation in regions associated with emotional control in the frontal and cingulate cortices (ie, mOFC, MFC, and IC), independent of cognitive change. Furthermore, we found region-specific associations between amyloid accumulation and increasing depressive symptoms, both independent of and in synergy with cognitive performance, in older adults before the stage of cognitive decline. This suggests there may be different factors (eg, regional amyloid accumulation) vs the reaction to cognitive changes independently influencing longitudinal trajectories in depressive symptoms. These results shed potential light on the neurobiology of depression in older individuals and underscore the importance of monitoring new and increasing affective symptoms in addition to cognitive changes in older adults presenting in psychiatry clinics and when screening for AD. Furthermore, they indicate that at least in a subset of older adults, the association between depression and dementia risk may, in part, be modified by regional amyloid accumulation. Together, findings provide support for early neuropsychiatric symptoms of dementia and highlight potential early opportunities for intervention.

## Notes

Supplement 1.
eMethods.
**eResults.** Sensitivity Analyses **eFigure 1.** Boxplot of Geriatric Depression Scale (GDS) Scores by Study Year, With Jitter Added as an Overlay to Demonstrate the Density of Individual Cases **eFigure 2.** PiB (Amyloid) Change/Slope Maps Created Using OLS Regression With Time as Only Factor at Each Vertex and Mixed-Effects Models Run at Each Vertex **eTable 1.** Baseline Demographics and Clinical Characteristics of the Subset of Participants From the Whole Sample Who Were Lost to Follow-Up Over the Course of the Study **eTable 2.** Results of Separate Linear Mixed-Effects Models in Which Regional Amyloid (PiB Slope) and Cognitive Performance Slope (PACC Slope) Were Interactive With Longitudinal Depressive Symptoms (GDS Score), Adjusting for Age, Sex, and Education **eTable 3.** Sensitivity Analyses Repeating the Primary Linear Mixed-Effects Model of Interest, With Regional Baseline PiB as a Covariate **eTable 4.** Sensitivity Analyses Repeating the Primary Linear Mixed-Effects Model of Interest, With a Modified GDS Score **eTable 5.** Sensitivity Analyses Repeating the Linear Mixed Effects Models Using PACC and PiB Slopes, with ADI as an Additional Covariate **eTable 6.** Sensitivity Analyses Repeating the Linear Mixed-Effects Models Using PACC (Cognition) and PiB (Amyloid) Slopes, With Self-Reported History of Depression as a Covariate **eTable 7.** Sensitivity Analyses Repeating the Linear Mixed-Effects Models using PACC (Cognition) and PiB (Amyloid) Slopes, With Self-Reported Use of Common Antidepressant Medications as Additional Covariates

Supplement 2.
Data Sharing Statement
