# Supplementary material for: Change in Depressive Symptoms and Longitudinal Regional Amyloid Accumulation in Unimpaired Older Adults
Source: JAMA Netw Open. 2024 Aug 29;7(8):e2427248. doi: 10.1001/jamanetworkopen.2024.27248 (PMC11362871; doi:10.1001/jamanetworkopen.2024.27248)
Supplement: Supplement 1. — eMethods. eResults. Sensitivity Analyses eFigure 1. Boxplot of Geriatric Depression Scale (GDS) Scores by Study Year, With Jitter Added as an Overlay to Demonstrate the Density of Individual Cases eFigure 2. PiB (Amyloid) Change/Slope Maps Created Using OLS Regression With Time as Only Factor at Each Vertex and Mixed-Effects Models Run at Each Vertex eTable 1. Baseline Demographics and Clinical Characteristics of the Subset of Participants From the Whole Sample Who Were Lost to Follow-Up Over the Course of the Study eTable 2. Results of Separate Linear Mixed-Effects Models in Which Regional Amyloid (PiB Slope) and Cognitive Performance Slope (PACC Slope) Were Interactive With Longitudinal Depressive Symptoms (GDS Score), Adjusting for Age, Sex, and Education eTable 3. Sensitivity Analyses Repeating the Primary Linear Mixed-Effects Model of Interest, With Regional Baseline PiB as a Covariate eTable 4. Sensitivity Analyses Repeating the Primary Linear Mixed-Effects Model of Interest, With a Modified GDS Score eTable 5. Sensitivity Analyses Repeating the Linear Mixed Effects Models Using PACC and PiB Slopes, with ADI as an Additional Covariate eTable 6. Sensitivity Analyses Repeating the Linear Mixed-Effects Models Using PACC (Cognition) and PiB (Amyloid) Slopes, With Self-Reported History of Depression as a Covariate eTable 7. Sensitivity Analyses Repeating the Linear Mixed-Effects Models using PACC (Cognition) and PiB (Amyloid) Slopes, With Self-Reported Use of Common Antidepressant Medications as Additional Covariates [file jamanetwopen-e2427248-s001.pdf]

Supplemental Online Content

Munro CE, Farrell M, Hanseeuw B, et al. Change in depressive symptoms and longitudinal regional amyloid accumulation in unimpaired older adults. *JAMA Netw Open.* 2024;7(8):e2427248. doi:10.1001/jamanetworkopen.2024.27248

eMethods.

eResults. Sensitivity Analyses

eFigure 1. Boxplot of Geriatric Depression Scale (GDS) Scores by Study Year, With Jitter Added as an Overlay to Demonstrate the Density of Individual Cases

eFigure 2. PiB (Amyloid) Change/Slope Maps Created Using OLS Regression With Time as Only Factor at Each Vertex and Mixed-Effects Models Run at Each Vertex

eTable 1. Baseline Demographics and Clinical Characteristics of the Subset of Participants From the Whole Sample Who Were Lost to Follow-Up Over the Course of the Study

eTable 2. Results of Separate Linear Mixed-Effects Models in Which Regional Amyloid (PiB Slope) and Cognitive Performance Slope (PACC Slope) Were Interactive With Longitudinal Depressive Symptoms (GDS Score), Adjusting for Age, Sex, and Education

eTable 3. Sensitivity Analyses Repeating the Primary Linear Mixed-Effects Model of Interest, With Regional Baseline PiB as a Covariate

eTable 4. Sensitivity Analyses Repeating the Primary Linear Mixed-Effects Model of Interest, With a Modified GDS Score

eTable 5. Sensitivity Analyses Repeating the Linear Mixed Effects Models Using PACC and PiB Slopes, with ADI as an Additional Covariate

eTable 6. Sensitivity Analyses Repeating the Linear Mixed-Effects Models Using PACC (Cognition) and PiB (Amyloid) Slopes, With Self-Reported History of Depression as a Covariate

eTable 7. Sensitivity Analyses Repeating the Linear Mixed-Effects Models using PACC (Cognition) and PiB (Amyloid) Slopes, With Self-Reported Use of Common Antidepressant Medications as Additional Covariates

This supplemental material has been provided by the authors to give readers additional information about their work.

© 2024 Munro CE et al. *JAMA Network Open.*

eMethods.

MRI and PiB-PET Acquisition and Processing and Additional Statistical Analysis Information (Mathematical Notation; Exploratory Analysis):

MRI and PiB-PET Acquisition and Processing:

Magnetic resonance imaging (MRI) was performed on a 3T Tim Trio (Siemens) and included a magnetization-prepared rapid gradient-echo (MPRAGE) processed with Freesurfer as described previously to identify grey-white and pial surfaces to permit ROI parcellation[46].

PiB-PET images were acquired with a 60-minute dynamic acquisition starting directly post-injection. Images were processed as previously reported[17] using an in-house pipeline designed to favor longitudinal reliability[17,47–52]. To limit error related to independent MR-PET co-registration, HABS PIB-PET scans were realigned to the baseline PET image and co-registered to an averaged MRI across all time points using FreeSurfer v6.0. To limit change over time due to the reference tissue, we utilized a composite reference region (cerebellum and eroded cortical white matter) with better stability over time[17,48] resulting in lower distribution volume ratio (DVR) values than are typically observed with cross-sectional amyloid PET approaches. Partial volume correction (PVC) methods were not used for these analyses, as PVC methods (particularly geometric transfer matrix PVC) have been shown to result in less precise measurements using longitudinal data, even when using longitudinally-stabilized approaches[52]. Additional exploratory vertex-wise analyses were also conducted with PIB DVR images projected onto the fsaverage surfaces from FreeSurfer and surface-smoothed to 8mm FWHM Gaussian kernel. PIB change/slope maps were created using OLS regression with time as the only predictor. Mixed-effects models were run at each vertex using the same model structure as primary analyses (i.e., the interaction between PiB and time predicting longitudinal GDS scores)

© 2024 Munro CE et al. *JAMA Network Open.*

with effect-size surfaces created for all fixed effects. Both vertex-wise maps reflect the t-values for the interaction term between PiB and time predicting longitudinal GDS.

Additional Statistical Analysis Information:

Simplified descriptions of statistical models were described in the main methods and were noted in the main manuscript tables for ease of readability; for example our primary model:

Longitudinal GDS ~ Regional PiB slope*time + (age, sex, education)*time

To provide additional statistical clarity on the models utilized, the mathematical notation of the regression equation model has also been provided below:

$$GDS_{ij} = \beta_0 + \beta_1 * time_{ij} + \beta_2 * PIBslope_i + \beta_3 * age_i + \beta_4 * sex_i + \beta_5 * education_i + \beta_6 * time_{ij} * PIBslope_i + \beta_7 * time_{ij} * age_i + \beta_8 * time_{ij} * sex_i + \beta_9 * time_{ij} * education_i + b_{0,i} + b_{1,i} * time_{ij} + e_{ij}$$

Exploratory Analysis (3-way interaction between PiB slope, PACC slope, and time):

In primary models, the 'PiB slope*time' term was the predictor of interest relative to outcome longitudinal GDS. In secondary models, 'PACC-slope*time' was added to models a predictor. Exploratory models including the 3-way interaction: (PiB slope*time)*PACC slope were also run to further probe relationships among longitudinal depression scores, PiB slope, cognition and time. Of particular interest were brain regions for which the regional PiB slope*time remained a significant predictor of longitudinal GDS, in the absence of significant 3-way interaction (regional PiB slope*PACC slope*time) (as seen in the mOFC and IC). This suggests that the relationship between depressive symptoms and PiB may not be solely

© 2024 Munro CE et al. *JAMA Network Open.*

dependent on cognition but, rather, represent a separate pathological etiology (e.g. amyloid accumulation in regions subserving emotional control).

eResults. Sensitivity Analyses

As described in the body of the manuscript, results of primary models were similar when using modified GDS scores with cognitive items removed, and when state ADI scores were included as a covariate (**Supplementary Tables 2-3**). National ADI scores were a significant covariate in models using PiB slopes in the lOFC, MFC, SFC, IC, and amygdala ROIs to predict longitudinal GDS scores, though these effects were small and the primary predictors (i.e., PiB and PACC slopes) remained significant (**Supplementary Table 4**).

Participants were asked about the use of selective serotonin reuptake inhibitors (SSRI), serotonin-norepinephrine reuptake inhibitors (SNRI), and serotonin antagonist and reuptake inhibitors (SARI)). Approximately 19% of participants reported a history of antidepressant use, but no participants reported a history of SARI use. Neither self-reported history of depression nor history of SSRI or SNRI use were significant covariates when added to primary models and relationships between PiB slope in the mOFC and IC remained significant with these additional covariates present; however, the relationship between PACC slopes and longitudinal GDS scores was suppressed when these psychiatric history variables were included in models (**Supplementary Tables 5-6**).

© 2024 Munro CE et al. *JAMA Network Open.*

eFigure 1. Boxplot of Geriatric Depression Scale (GDS) Scores by Study Year, With Jitter Added as an Overlay to Demonstrate the Density of Individual Cases (n=154).

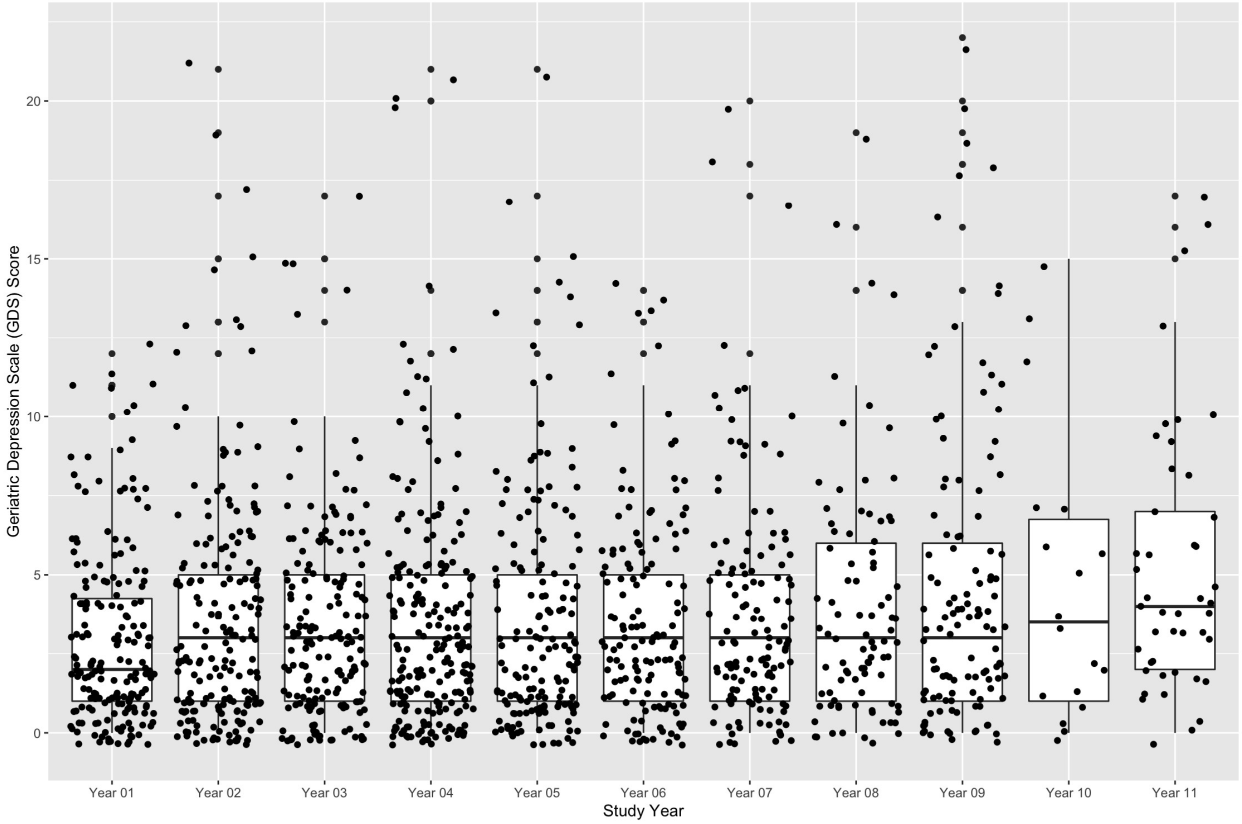

© 2024 Munro CE et al. *JAMA Network Open.*

eFigure 2. PiB (Amyloid) Change/Slope Maps Created Using OLS Regression With Time as Only Factor at Each Vertex and Mixed-Effects Models Run at Each Vertex (p<0.01; model: longitudinal GDS ~ amyloid slope*time + PACC slope*time + (sex + edu + age)*time). This map visualizes the results (t score, PiB slope region*time) of linear mixed-effects models such that regions with a greater t score (red) represent areas in which greater depressive symptoms are associated with greater amyloid levels.

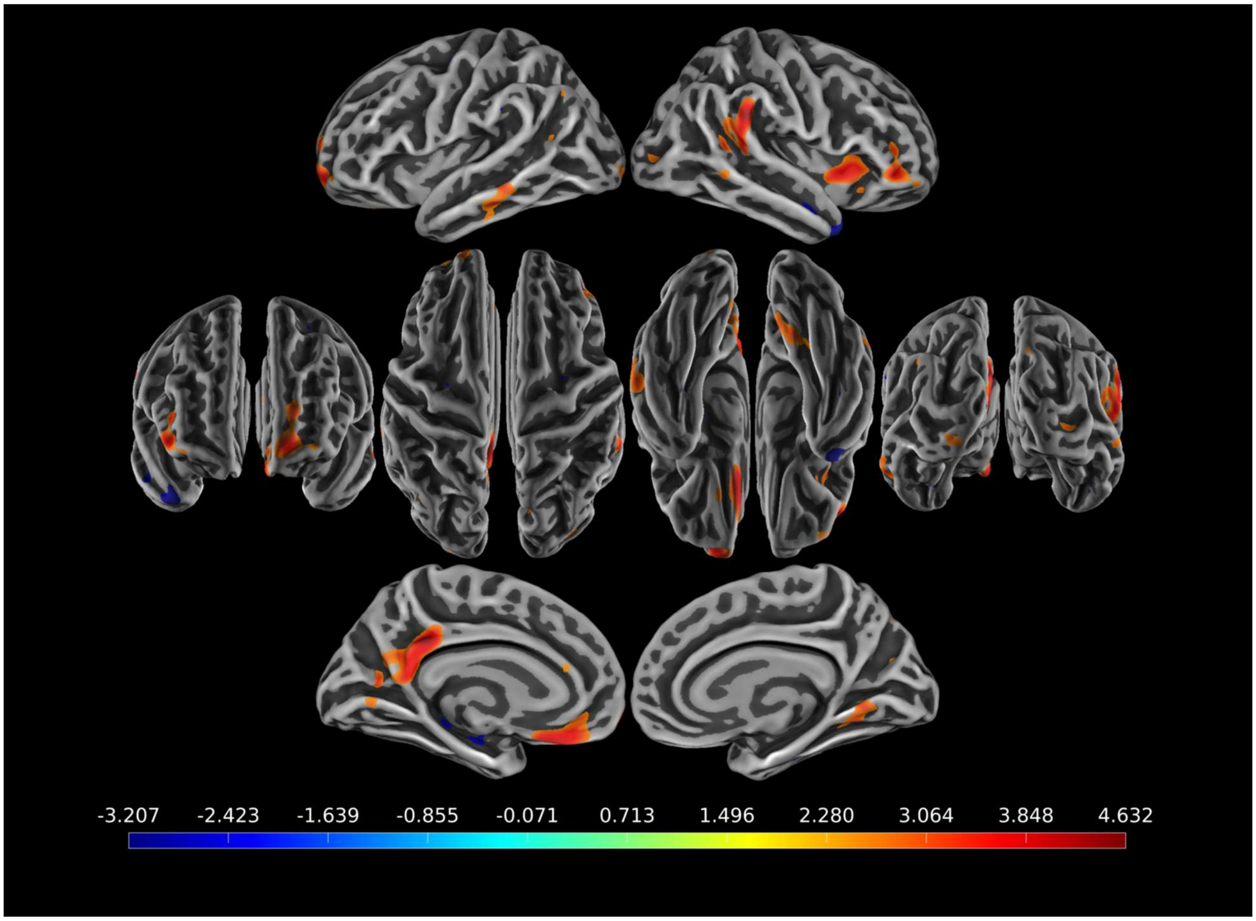

© 2024 Munro CE et al. *JAMA Network Open.*

eTable 1. Baseline Demographics and Clinical Characteristics of the Subset of Participants From the Whole Sample Who Were Lost to Follow-Up Over the Course of the Study (n=17)

T-tests and chi-squared tests were used to determine whether demographic data in this subset differed from that of the whole sample. While the subset of individuals lost to follow-up had slightly lower baseline PACC (cognition) scores and, as expected, fewer years of total follow-up compared to the whole sample, demographic data were otherwise similar between groups.

**Demographic Variable (n=17)**	**Mean or Percentage**	**SD**	**Range**	**Comparison to whole sample**	
				**t-score or χ^2**	**p-value**
Age (years)	74.1	8.5	65.8 – 89.3	0.89	0.38
Sex (% female)	52.9	---	---	0.42	0.52
Race (% white)	82.4	---	---	0.05	0.82
Ethnicity (% non-Hispanic)	100.0	---	---	1.87	0.17
Education (years)	15.2	3.9	6.0 – 20.0	0.86	0.39
Baseline GDS Score	3.2	0.6	0.0 – 8.0	0.14	0.89
Baseline PiB DVR (frontolateral retrosplenial aggregate)	0.8	0.03	0.8 – 0.9	0.00	0.99
Baseline PACC-5 Z-Score	-0.3	0.6	-1.7 – 0.5	2.26	**0.02**
Years of Follow Up	4.5	1.3	2.8 – 7.7	7.53	**<0.001**

Abbreviations: GDS, Geriatric Depression Scale 30 item; PiB, Pittsburgh Compound B; DVR, Distribution Volume Ratio; PACC-5, Preclinical Alzheimer Cognitive Composite - 5.

© 2024 Munro CE et al. *JAMA Network Open.*

eTable 2. Results of Separate Linear Mixed-Effects Models in Which Regional Amyloid (PiB Slope) and Cognitive Performance Slope (PACC Slope) Were Interactive With Longitudinal Depressive Symptoms (GDS Score), Adjusting for Age, Sex, and Education For models in which the 3-way interaction was not significant, we also included p-values for significant 2-way interactions between PiB slope*time and/or PACC slope*time in the last column. All 3-way-interaction p-values are adjusted for multiple comparisons using an FDR correction.

**Model:** longitudinal GDS ~ amyloid (regional PiB) slope*PACC slope*time + (sex + edu + age)*time				
	3-way interaction: PiB Slope*PACC slope*Time			Significant 2-way interactions
	β	**t (SE) [95%CI]**	**Adjusted p-value**	**(p-value)**
mOFC	-59.60	-1.79 (33.29) [-124.90, 5.69]	0.20	PiB slope*time **(0.002)**
lOFC	-14.25	-0.42 (33.71) [-80.37, 51.86]	0.67	PACC slope*time **(0.005)**
MFC	-118.68	-3.23 (36.78) [-190.82, -46.54]	**0.008**	---
SFC	-122.33	-3.12 (39.27) [-199.34, -45.31]	**0.008**	---
ACC	-44.14	-1.49 (29.69) [-102.37, 14.09]	0.28	---
IC	-31.30	-0.26 (37.22) [-104.31, 41.71]	0.46	PIB slope*time **(0.008)**
PCC	-35.95	-1.14 (31.47) [-97.67, 25.77]	0.39	
Amygdala	43.01	1.07 (40.34) [-36.13, 122.15]	0.39	PACC slope*time **(0.003)**

Abbreviations: GDS, Geriatric Depression Scale 30 item, β, beta/regression coefficient; SE, standard error; CI, confidence interval; edu, education; mOFC, medial orbitofrontal cortex; lOFC, lateral orbitofrontal cortex; MFC, middle frontal cortex; SFC, superior frontal cortex; ACC, anterior cingulate cortex; IC, isthmus

© 2024 Munro CE et al. *JAMA Network Open.*

cingulate cortex; PCC, posterior cingulate cortex; PiB, Pittsburgh Compound B; PACC, Preclinical Alzheimer Cognitive Composite-5.

© 2024 Munro CE et al. *JAMA Network Open.*

eTable 3. Sensitivity Analyses Repeating the Primary Linear Mixed-Effects Model of Interest, With Regional Baseline PiB as a Covariate

Longitudinal depressive symptoms (GDS score) were predicted by amyloid (PiB) slope in a region of interest, this time introducing regional baseline PiB as a covariate. Models were also adjusted for age, sex, and education. Results remain similar to primary analyses.

Model: longitudinal GDS ~ regional amyloid slope*time + baseline regional amyloid*time + (sex + edu + age)*time					
	β	t	SE	95%CI	p-value
mOFC	9.98	5.51	1.81	6.42 – 13.53	**<0.001**
lOFC	4.14	1.04	1.75	0.71 – 7.57	**0.02**
MFC	7.56	3.62	2.01	3.46 - 11.56	**<0.001**
SFC	1.71	0.87	1.96	-2.14 – 5.56	0.38
ACC	5.62	3.61	1.56	2.56 – 8.67	**<0.001**
IC	11.06	4.97	2.22	6.70 – 15.42	**<0.001**
PCC	3.60	2.04	1.77	0.14 – 7.06	**0.042**
Amygdala	-3.44	-1.56	2.21	-7.77 – 0.89	0.12

Abbreviations: GDS, Geriatric Depression Scale 30 item; β, beta/regression coefficient; SE, standard error; CI, confidence interval; edu, education; mOFC, medial orbitofrontal cortex; lOFC, lateral orbitofrontal cortex; MFC, middle frontal cortex; SFC, superior frontal cortex; ACC, anterior cingulate cortex; IC, isthmus cingulate cortex; PCC, posterior cingulate cortex; PiB, Pittsburgh Compound B.

© 2024 Munro CE et al. *JAMA Network Open.*

eTable 4. Sensitivity Analyses Repeating the Primary Linear Mixed-Effects Model of Interest, With a Modified GDS Score

Longitudinal depressive symptoms (GDS score) were predicted by amyloid (PiB) slope in a region of interest, this time using a modified GDS-30 score, in which cognitive items removed from the GDS score. Results remain similar to primary analyses.

Model: longitudinal GDS (without cognitive items) ~ amyloid slope*time + (sex + edu + age)*time					
	β	t	SE	95%CI	p-value
mOFC	8.77	5.37	1.63	5.56 – 11.98	**<0.001**
lOFC	2.95	2.06	1.43	0.13 – 5.76	**0.04**
MFC	6.65	3.47	1.92	2.89 – 10.41	**0.001**
SFC	1.23	0.68	1.80	-2.30 – 4.75	0.50
ACC	5.02	3.54	1.42	2.24 – 7.80	**<0.001**
IC	9.67	4.80	2.02	5.71 – 13.64	**<0.001**
PCC	3.51	2.23	1.58	0.42 – 6.60	**0.03**
Amygdala	-1.16	0.01	1.81	-4.95 – 2.64	0.55

Abbreviations: GDS, Geriatric Depression Scale 30 item; β, beta/regression coefficient; SE, standard error; CI, confidence interval; edu, education; mOFC, medial orbitofrontal cortex; lOFC, lateral orbitofrontal cortex; MFC, middle frontal cortex; SFC, superior frontal cortex; ACC, anterior cingulate cortex; IC, isthmus cingulate cortex; PCC, posterior cingulate cortex; PiB, Pittsburgh Compound B.

© 2024 Munro CE et al. *JAMA Network Open.*

eTable 5. Sensitivity Analyses Repeating the Linear Mixed Effects Models Using PACC and PiB Slopes, with ADI as an Additional Covariate

PACC and PiB slopes were used to predict longitudinal GDS scores with the log of national Area Deprivation Index (ADI) scores from 2021 as an additional covariate. Models were also adjusted for age, sex, and education. Increasing ADI scores indicate greater deprivation scores (ranged 0 -100, 100 being most deprived). National ADI score was a significant covariate in models looking at lOFC, MFC, SFC, IC, and amygdala; however, effect sizes of ADI were fairly small and the relationships seen in primary models (i.e., looking at PiB and/or PACC predictor variables) were unchanged. State ADI scores were also examined in separate models (without national ADI scores) and were not significant predictors of GDS across any of the models.

Model:
longitudinal GDS ~ regional amyloid slope*time + PACC slope*time + (sex + edu + age)*time + national ADI*time

Region	**Variable (Interaction with Time)**	**β**	**t**	**SE**	**95%CI**	**p-value**
mOFC	PiB slope	8.05	4.59	1.76	4.61 – 11.50	**<0.001**
	PACC slope	-1.55	-4.40	0.35	-2.24 – (-0.86)	**<0.001**
	ADI	-0.04	-1.81	0.02	-0.09 – 0.001	0.07
lOFC	PiB slope	1.10	0.70	1.56	-1.97 – 4.16	0.48
	PACC slope	-1.60	-4.49	0.36	-2.31 – (-)0.90	**<0.001**
	ADI	-0.05	-2.00	0.02	-0.09 – (-)0.001	**0.045**
MFC	PiB slope	4.42	2.12	2.08	0.33 – 8.50	**0.03**
	PACC slope	-1.55	-4.33	0.36	-2.25 – (-)0.85	**<0.001**
	ADI	-0.04	-1.97	0.02	-0.09 – 0.001	**0.049**
SFC	PiB slope	-0.27	-0.14	1.92	-4.04 – 3.49	0.89
	PACC slope	-1.65	-4.64	0.36	-2.34 – (-)0.95	**<0.001**

© 2024 Munro CE et al. *JAMA Network Open.*

	ADI	-0.05	-2.11	0.02	-0.09 – 0.001	**0.04**
ACC	PiB slope	4.16	2.73	1.52	1.17 – 7.15	**0.006**
	PACC slope	-1.52	-4.26	0.36	-2.22 – (-)0.82	**<0.001**
	ADI	-0.04	-1.94	0.02	-0.09 – 0.001	0.05
IC	PiB slope	10.21	4.60	2.22	5.86 – 14.57	**<0.001**
	PACC slope	-1.31	-3.66	0.36	-2.02 – (-)0.61	**<0.001**
	ADI	-0.04	-1.97	0.02	-0.09 – (-)0.001	**0.049**
PCC	PiB slope	10.35	4.66	2.22	5.99 – 14.71	**<0.001**
	PACC slope	-1.27	-3.53	0.36	-1.97 – (-)0.56	**<0.001**
	ADI	0.16	-1.40	0.03	-0.10 – 0.02	0.16
Amygdala	PiB slope	-0.39	-0.18	2.15	-4.61 – 3.82	0.86
	PACC slope	-1.64	-4.62	0.35	-2.33 – (-)0.94	**<0.001**
	ADI	-0.05	-2.08	0.02	-0.09 – (-)0.001	**0.038**

Abbreviations: GDS, Geriatric Depression Scale 30 item, β, beta/regression coefficient; SE, standard error; CI, confidence interval; edu, education; mOFC, medial orbitofrontal cortex; lOFC, lateral orbitofrontal cortex; MFC, middle frontal cortex; SFC, superior frontal cortex; ACC, anterior cingulate cortex; IC, isthmus cingulate cortex; PCC, posterior cingulate cortex; PiB, Pittsburgh Compound B; PACC, Preclinical Alzheimer Cognitive Composite-5; ADI, Area Deprivation Index.

eTable 6. Sensitivity Analyses Repeating the Linear Mixed-Effects Models Using PACC (Cognition) and PiB (Amyloid) Slopes, With Self-Reported History of Depression as a Covariate

PACC and PiB slopes were used to predict longitudinal GDS (depression) scores with self-reported history of depression as a covariate. Models were also adjusted for age, sex, and education. Depression history was not a significant predictor of longitudinal GDS scores in any model. PiB slopes remained a significant predictor in the mOFC and IC regions. Relationships between PACC slopes and longitudinal GDS scores were suppressed in models with depression history as a covariate.

Model:
longitudinal GDS ~ regional amyloid slope*time + PACC slope*time + (sex + edu + age)*time + depression history*time

Region	**Variable (Interaction with Time)**	**β**	**t**	**SE**	**95%CI**	**p-value**
mOFC	PiB slope	6.87	2.73	2.52	1.93 – 11.81	**0.007**
	PACC slope	0.03	0.06	0.53	-1.00 – 1.06	0.95
	Depression Hx	0.12	1.69	0.07	-0.02 – 0.25	0.09
lOFC	PiB slope	2.26	0.97	2.34	-1.97 – 4.16	0.33
	PACC slope	0.06	0.12	0.53	-2.31 – (-)0.90	0.91
	Depression Hx	0.11	1.60	0.07	-0.09 – (-)0.001	0.11
MFC	PiB slope	0.46	0.14	3.25	-5.91 – 6.83	0.89
	PACC slope	0.05	0.09	0.53	-1.00 – 1.09	0.93
	Depression Hx	0.11	1.64	0.07	-0.02 – 0.25	0.10
SFC	PiB slope	0.15	0.05	3.35	-6.43 – 6.74	0.96
	PACC slope	0.04	0.07	0.53	-1.00 – 1.07	0.95

© 2024 Munro CE et al. *JAMA Network Open.*

	Depression Hx	0.11	1.63	0.07	-0.02 – 0.25	0.10
ACC	PiB slope	3.68	1.61	2.29	-0.81 – 8.18	0.11
	PACC slope	0.12	0.22	0.53	-0.92 – 1.15	0.83
	Depression Hx	0.12	1.79	0.07	-0.01 – 0.26	0.08
IC	PiB slope	10.97	3.29	3.33	4.42 – 17.51	**0.001**
	PACC slope	0.14	0.26	0.53	-0.89 – 1.17	0.79
	Depression Hx	0.13	1.87	0.07	-0.01 – 0.26	0.06
PCC	PiB slope	4.79	1.70	2.82	-0.75 – 10.34	0.09
	PACC slope	-0.16	0.30	0.53	-0.88 – 1.20	0.76
	Depression Hx	0.11	1.68	0.07	-0.02 – 0.25	0.09
Amygdala	PiB slope	1.32	0.42	3.18	-4.92 – 7.57	0.68
	PACC slope	0.004	0.01	0.35	-1.04 – 1.05	0.99
	Depression Hx	0.11	0.07	0.07	-0.03 – 0.24	0.11

Abbreviations: GDS, Geriatric Depression Scale 30 item, β, beta/regression coefficient; SE, standard error; CI, confidence interval; edu, education; mOFC, medial orbitofrontal cortex; lOFC, lateral orbitofrontal cortex; MFC, middle frontal cortex; SFC, superior frontal cortex; ACC, anterior cingulate cortex; IC, isthmus cingulate cortex; PCC, posterior cingulate cortex; PiB, Pittsburgh Compound B; PACC, Preclinical Alzheimer Cognitive Composite-5; ADI, Area Deprivation Index; Depression Hx, Depression History.

© 2024 Munro CE et al. *JAMA Network Open.*

eTable 7. Sensitivity Analyses Repeating the Linear Mixed-Effects Models using PACC (Cognition) and PiB (Amyloid) Slopes, With Self-Reported Use of Common Antidepressant Medications as Additional Covariates

PACC and PiB slopes were used to predict longitudinal GDS (depression) scores with self-reported use of common antidepressant medications (i.e., selective serotonin reuptake inhibitors (SSRI) and serotonin and norepinephrine reuptake inhibitors (SNRI)) as additional covariates. Models were also adjusted for age, sex, and education. Separate models were run using each type of antidepressant as a covariate (i.e., the model using mOFC PiB slope and PACC slope to predict longitudinal GDS with only history of SSRI use as a covariate was run first, then the same model with only history of SNRI use as a covariate was run). Participants were also asked about use of serotonin antagonist and reuptake inhibitors (SARI), but no participants in this sample reported a history of SARI use. Results were similar to sensitivity analyses performed using history of depression as a covariate. Neither SSRI nor SNRI use was a significant predictor of longitudinal GDS scores in any model. PiB slopes remained a significant predictor in the mOFC and IC regions. Relationships between PACC slopes and longitudinal GDS scores were suppressed in models with historical use of either antidepressant medication type as a covariate.

**Model:** longitudinal GDS ~ regional amyloid slope*time + PACC slope*time + (sex + edu + age)*time + antidepressant (SSRI or SNRI) use*time						
Region	**Variable (Interaction with Time)**	**β**	**t**	**SE**	**95%CI**	**p-value**
mOFC	PiB slope	6.70	2.66	2.52	1.75 – 11.65	**0.008**
	PACC slope	-0.06	-0.11	0.52	-1.08 – 0.97	0.91

© 2024 Munro CE et al. *JAMA Network Open.*

	SSRI use	0.10	1.24	0.08	-0.06 – 0.25	0.22
	PiB slope	6.39	2.54	2.51	1.46 – 11.32	**0.01**
	PACC slope	-0.02	-0.03	0.52	-1.05 – 1.01	0.97
	SNRI use	-0.02	-0.11	0.19	-0.39 – 0.35	0.92
lOFC	PiB slope	2.47	1.06	2.33	-2.10 – 7.05	0.29
	PACC slope	-0.02	-0.03	0.52	-1.05 – 1.01	0.97
	SSRI use	-0.08	1.06	0.08	-0.07 – 0.24	0.29
	PiB slope	2.33	1.00	2.32	-2.23 – 6.88	0.32
	PACC slope	0.01	0.03	0.53	-1.02 – 1.05	0.98
	SNRI use	-0.02	-0.12	0.19	-0.39 – 0.35	0.90
MFC	PiB slope	0.18	0.06	3.25	-6.21 – 6.57	0.96
	PACC slope	-0.03	-0.07	0.53	-1.07 – 1.00	0.95
	SSRI use	0.08	0.98	0.08	-0.08 – 0.23	0.33
	PiB slope	-0.25	-0.08	3.24	-6.61 – 6.12	0.94
	PACC slope	-0.01	-0.02	0.53	-1.05 – 1.03	0.99
	SNRI use	-0.02	-0.12	0.19	-0.40 – 0.35	0.91
SFC	PiB slope	-0.002	-0.001	3.36	-6.60 – 6.60	0.99
	PACC slope	-0.04	-0.08	0.53	-1.07 – 0.99	0.94
	SSRI use	0.08	0.97	0.08	-0.08 – 0.23	0.33
	PiB slope	-0.40	-0.12	3.33	-6.94 – 6.14	0.90
	PACC slope	-0.01	-0.03	0.53	-1.05 – 1.02	0.98
	SNRI use	-0.02	-0.12	0.19	-0.39 – 0.35	0.90
ACC	PiB slope	2.75	1.20	2.29	-1.74 – 7.24	0.23
	PACC slope	0.01	0.02	0.53	-1.02 – 1.05	0.98
	SSRI use	0.09	1.15	0.08	-0.06 – 0.25	0.25
	PiB slope	2.37	1.05	2.27	-2.08 – 6.82	0.30
	PACC slope	0.04	0.08	0.53	-0.99 – 1.08	0.94
	SNRI use	-0.03	-0.14	0.19	-0.40 – 0.34	0.89
IC	PiB slope	10.87	3.18	3.42	4.16 – 17.59	**0.002**
	PACC slope	0.03	0.06	0.52	-0.99 – 1.06	0.95
	SSRI use	0.15	1.82	0.08	-0.01 – 0.31	0.07

© 2024 Munro CE et al. *JAMA Network Open.*

	PiB slope	9.09	2.76	3.30	2.61 – 15.56	**0.006**
	PACC slope	0.06	0.12	0.52	-0.00 – 0.02	0.90
	SNRI use	-0.01	-0.05	0.19	-0.38 – 0.36	0.96
PCC	PiB slope	5.11	1.81	2.83	-0.043 – 10.66	0.07
	PACC slope	0.08	0.15	0.53	-0.96 – 1.12	0.88
	SSRI use	0.10	1.21	0.08	-0.06 – 0.25	0.23
	PiB slope	4.63	1.65	2.81	-0.88 – 10.13	0.10
	PACC slope	0.10	0.20	0.53	-0.94 – 1.14	0.85
	SNRI use	-0.01	-0.05	0.19	-0.38 – 0.36	0.96
Amygdala	PiB slope	1.76	0.55	3.18	-4.48 – 8.00	0.58
	PACC slope	-0.08	-0.16	0.53	-1.13 – 0.96	0.88
	SSRI use	0.08	0.96	0.08	-0.08 – 0.23	0.34
	PiB slope	2.12	0.67	3.17	-4.11 – 8.34	0.50
	PACC slope	-0.06	-0.11	0.53	-1.10 – 0.99	0.91
	SNRI use	-0.03	-0.14	0.19	-0.40 – 0.35	0.89

Abbreviations: GDS, Geriatric Depression Scale 30 item, β, beta/regression coefficient; SE, standard error; CI, confidence interval; edu, education; mOFC, medial orbitofrontal cortex; lOFC, lateral orbitofrontal cortex; MFC, middle frontal cortex; SFC, superior frontal cortex; ACC, anterior cingulate cortex; IC, isthmus cingulate cortex; PCC, posterior cingulate cortex; PiB, Pittsburgh Compound B; PACC, Preclinical Alzheimer Cognitive Composite-5; SSRI, Selective Serotonin Reuptake Inhibitor; SNRI, Serotonin Norepinephrine Reuptake Inhibitor.
