# Supplementary material for: Change in Depressive Symptoms and Longitudinal Regional Amyloid Accumulation in Unimpaired Older Adults
Source: JAMA Netw Open. 2024 Aug 29;7(8):e2427248. doi: 10.1001/jamanetworkopen.2024.27248 (PMC11362871; doi:10.1001/jamanetworkopen.2024.27248)
Supplement: Supplement 2. — Data Sharing Statement [file jamanetwopen-e2427248-s002.pdf]

Data Sharing Statement

Munro. Change in Depressive Symptoms and Longitudinal Regional Amyloid Accumulation in Unimpaired Older Adults. *JAMA Netw Open*. Published August 29, 2024. doi:10.1001/jamanetworkopen.2024.27248

Data
Data available: Yes
Data types: Deidentified participant data
How to access data: Data sharing requests will follow the pre-existing format of the Harvard Aging Brain Study data release. Interested investigators can visit the Harvard Aging Brain Study website (https://habs.mgh.harvard.edu/researchers/request-data/) to submit a data request, and/or email habsdata@mgh.harvard.edu for more information.
When available: With publication

Supporting Documents
Document types: None

Additional Information
Who can access the data: To request data, an individual must be either an academic or industry researcher. Completing a data use agreement is required and all requests must be approved by the data team, the principal investigators of all three studies, and the principal investigator of the proposed project. The data use agreement exists on the Harvard Aging Brain Study website linked above and includes information about the researcher requesting the data, their institution and contact information, information about the proposed research (including study name), and what specific data (i.e., specific imaging scans or spreadsheets, clinical measures, questionnaires, etc.) are needed.
Types of analyses: Data use agreements must include the specific analyses planned and must be approved by the Harvard Aging Brain Study data analytics team.
Mechanisms of data availability: To request data, an individual must be either an academic or industry researcher. Completing a data use agreement is required and all requests must be approved by the data team, the principal investigators of all three studies, and the principal investigator of the proposed project. The data use agreement exists on the Harvard Aging Brain Study website linked above and includes information about the researcher requesting the data, their institution and contact information, information about the proposed research (including study name), and what specific data (i.e., specific imaging scans or spreadsheets, clinical measures, questionnaires, etc.) are needed. All data is deidentified upon initial entry into the database and any researchers (including the principal investigator of the proposed project) will only have access to deidentified data. Deidentification of data includes blinded participant identification numbers and blinded collection/birth dates (i.e., any dates are offset from the actual date by a random number for each participant that stays consistent throughout all visits). Any identifiable data is only disseminated on a need-to-know basis (e.g., notification of abnormal imaging results) with as little information disclosed as necessary to the fewest individuals possible to protect participant privacy. Data sharing requests and processing of data use agreements will be acknowledged and completed as soon as possible by the Harvard Aging Brain Study data team. There is currently no time limit or proposal limit for submission of data sharing requests or use agreements. If a data sharing request is approved and an agreement is signed, the Harvard Aging Brain Study data team will be in contact with approved investigators via email on an individual basis to determine the best or most appropriate data sharing modality.
